# Dengue virus dominates lipid metabolism modulations in *Wolbachia*-coinfected *Aedes aegypti*

**DOI:** 10.1038/s42003-020-01254-z

**Published:** 2020-09-18

**Authors:** Cassandra Koh, M. Nurul Islam, Yixin H. Ye, Nunya Chotiwan, Barbara Graham, John T. Belisle, Konstantinos A. Kouremenos, Saravanan Dayalan, Dedreia L. Tull, Stephan Klatt, Rushika Perera, Elizabeth A. McGraw

**Affiliations:** 1 School of Biological Sciences, Monash University, Clayton, VIC 3800 Australia; 2 Department of Microbiology, Immunology and Pathology, Colorado State University, Fort Collins, CO 80523 USA; 3 Metabolomics Australia, Bio21 Institute of Molecular Sciences and Biotechnology, University of Melbourne, Parkville, VIC 3010 Australia; 4 Department of Biochemistry and Molecular Biology, Bio21 Institute of Molecular Sciences and Biotechnology, University of Melbourne, Parkville, VIC 3010 Australia; 5 The Florey Institute of Neuroscience and Mental Health, University of Melbourne, Parkville, VIC 3052 Australia; 6 Department of Entomology, Center for Infectious Disease Dynamics, Huck Institutes of the Life Sciences, Pennsylvania State University, University Park, PA 16801 USA

**Keywords:** Biochemical networks, Pathogens

## Abstract

Competition between viruses and *Wolbachia* for host lipids is a proposed mechanism of *Wolbachia*-mediated virus blocking in insects. Yet, the metabolomic interaction between virus and symbiont within the mosquito has not been clearly defined. We compare the lipid profiles of *Aedes aegypti* mosquitoes bearing mono- or dual-infections of the *Wolbachia w*Mel strain and dengue virus serotype 3 (DENV3). We found metabolic signatures of infection-induced intracellular events but little evidence to support direct competition between *Wolbachia* and virus for host lipids. Lipid profiles of dual-infected mosquitoes resemble those of DENV3 mono-infected mosquitoes, suggesting virus-driven modulation dominates over that of *Wolbachia*. Interestingly, knockdown of key metabolic enzymes suggests cardiolipins are host factors for DENV3 and *Wolbachia* replication. These findings define the *Wolbachia*-DENV3 metabolic interaction as indirectly antagonistic, rather than directly competitive, and reveal new research avenues with respect to mosquito × virus interactions at the molecular level.

## Abstract (web summary)

Koh, Islam, Ye et al. describe lipid profiles of *Aedes aegypti* mosquitoes bearing mono- or dual-infections of *Wolbachia* (*w*Mel) and dengue virus serotype 3 (DENV3), finding that virus modulation dominates the dual-infection lipid profile and that cardiolipins support DENV3 and *Wolbachia* replication. This study suggests that direct competition for lipids do not underlie *Wolbachia*-mediated virus blocking.

## Introduction

*Wolbachia* is a ubiquitous intracellular bacterial symbiont of insects^1^. Maternally inherited, *Wolbachia* spreads and persists in host populations by manipulating host reproductive biology. Some insects harbouring *Wolbachia* are protected against viral pathogens, including arthropod-borne viruses. In naturally-infected *Drosophila melanogaster*, *Wolbachia* inhibits the replication of RNA viruses, leading to lower mortality in virus-infected flies^2,3^. Similarly, native *Wolbachia* in the mosquito vector *Culex quinquefasciatus* suppresses the replication of West Nile (WNV), chikungunya, and La Crosse viruses^4^. The ability to spread into insect populations via vertical transmission and to suppress replication of viral pathogens make *Wolbachia* an attractive method of intervention against mosquito-transmitted viral diseases, such as dengue viruses (DENVs)^5^. Dengue fever is a highly prevalent arboviral disease wherever the *Aedes aegypti* mosquito vector is present^6^. The *Wolbachia w*Mel strain was isolated from its native *D. melanogaster* host and transinfected into *A. aegypti* mosquitoes, which do not naturally carry the symbiont^5,7,8^. Mosquitoes bearing *w*Mel were then released in a series of field trials in Australia where dengue outbreaks are common. Years later, the bacterium has stably introgressed into wild mosquito populations at high frequencies^9,10^ and the intervention has eliminated local transmission of DENVs within the region^10^.

Despite the rising popularity of *Wolbachia* intervention, the precise molecular mechanisms behind the viral blocking phenotype remain unknown. Although viral blocking likely results from multiple complementary mechanisms, previous studies propose two main hypotheses^11–13^: immune priming, which reduces the success of secondary infections by invading pathogens^7,14–16^, and competition for limited host resources such as amino acids, lipids, and intracellular space^17–20^.

Throughout the infection cycle of DENVs, interactions with cellular lipids have been reported at multiple stages^21^. Zaitseva et al.^22^ showed that virus release into the cytosol requires fusion of the viral lipid envelope with endosomal membranes enriched in the anionic lipid bis(monoacylglycero)phosphate. DENVs replicate in intracellular structures derived from extensive remodeling of the endoplasmic reticulum (ER) membrane. These membrane structures house viral RNA replication factories and protect viral RNA from detection by pathogen recognition receptors^19,23,24^. DENVs are also speculated to increase the adenosine triphosphate (ATP) production in cells to fulfil the energetic demands of viral replication by increasing the availability of fatty acids for mitochondrial breakdown^25^. Chotiwan et al.^26^ found that DENV2 infection caused perturbations in the lipid profiles of mosquito midguts, which varied across early-, mid-, and late-stage infections. Taken together, lipid abundance and composition at the cellular and tissue level are critical for successful replication of DENVs within human and mosquito hosts.

Like most endosymbionts, *Wolbachia* proliferation in their natural *Drosophila* host was reported to be tightly associated with nutritional environment^27^. Molloy et al.^28^ showed that infection by two *Wolbachia* strains alters the lipidome of *A. albopictus* cells. The authors noted a depletion in sphingolipids, diacylglycerols, and phosphatidylcholines, which were also the lipid classes enriched during DENV2 replication in *A. albopictus* cells^29^. The strongest evidence implicating lipid competition as a mechanism of *Wolbachia* blocking comes from two studies demonstrating that supplementation of cholesterol compromises *Wolbachia*-induced blocking against virus replication^17,30^. When *Drosophila* flies harboring the *Wolbachia* strains *w*MelPop and *w*MelCS were reared on diets supplemented with cholesterol, *Wolbachia*-mediated virus blocking diminished in these flies as measured by survival and virus accumulation^17^. Another strain, *w*Stri, interferes with entry of Zika virus into *A. albopictus* mosquito cells. Similarly, the effect of this interference decreased when the cell growth medium was supplemented with cholesterol^30^.

It is apparent that interaction between *Wolbachia* and DENVs could occur at multiple points across the host metabolic landscape and that some lipid classes may play more critical roles than others. Identifying these interaction points will allow more definitive testing of whether lipids are involved with *Wolbachia*-mediated viral blocking. To this end, we compare the lipid profiles of mosquitoes to identify lipids altered by mono- or dual-infection of *Wolbachia* and dengue virus serotype 3 (DENV3) using a geographically pair-matched *A. aegypti* colony and virus strain. Our data showed little evidence for direct lipid competition and instead points toward *Wolbachia*-induced perturbations that are disadvantageous for DENV3 replication. Using a combination of our own lipid profile data and lipid metabolism-related genes reported in past studies, we sought to validate the relevance of certain lipid classes through genetic manipulation of their biosynthetic pathways. Disruption of the cardiolipin production through gene knockdown was detrimental to replication of both DENV3 and *Wolbachia* in mono-infections. To our knowledge, this is the first report of any link between cardiolipins (CLs) and replication of DENVs. Given the function of CLs in the inner mitochondrial membrane, this reinforces the importance of mitochondrial function for the replication of DENVs^31,32^.

## Results

### Infection-modulated lipid profiles

To examine *Wolbachia-* and DENV3-induced lipid modulation in *A. aegypti*, we used liquid chromatography–mass spectrometry (LC–MS) to compare the lipid profiles of mosquitoes harboring mono- or dual-infections against that of naive mosquitoes. An overview of the experimental design is depicted in Supplementary Fig. 1. In total, 1199 lipid molecules were detected. Following statistical analyses, lipids with abundances significantly altered by infection status were putatively identified using Human Metabolome Database (HMDB) and LIPID Metabolites and Pathways Strategy (LIPID MAPS) databases. Further compound validation was achieved by interpreting MS/MS spectra data when available. Representative MS/MS spectra for each lipid class identified are shown in Supplementary Figs. 2–7.

DENV3 mono-infection significantly modulated 47 lipids (Student’s two-sided *t* test, Benjamini–Hochberg (BH)-adjusted *p* < 0.05) (Supplementary Data 1, PCA shown in Supplementary Fig. 8). Remarkably, all these lipids were elevated in DENV3 mono-infected mosquitoes relative to naive mosquitoes (log_2_ fold change > 0). Ten lipids were putatively identified to Metabolomics Standard Initiative (MSI) level 2 and another 26 to MSI level 3. *Wolbachia* mono-infection significantly modulated the abundances of only eight lipids (Student’s two-sided *t* test, BH-adjusted *p* < 0.05) (Supplementary Data 2, PCA shown in Supplementary Fig. 8), with three lipids putatively identified to MSI level 2 and another three to MSI level 3. These eight lipids were mildly depleted in *Wolbachia* mono-infected mosquitoes relative to naive mosquitoes (log_2_ fold change < 0).

**Fig. 1:**
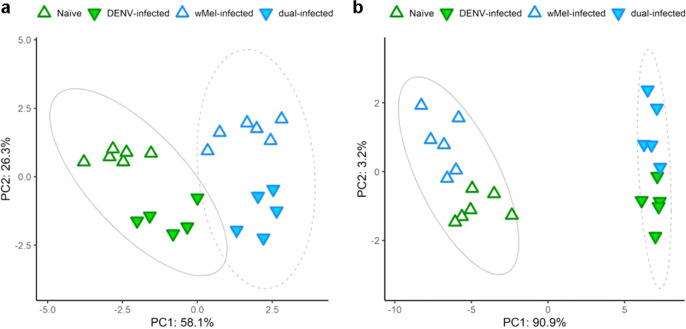
DENV- and *Wolbachia*-modulated lipids show evidence of interaction. Principle components analyses based on **a** DENV-modulated lipids or **b** *Wolbachia*-modulated lipids as variables reveal segregation of samples by their infection status. Each data point represents one mosquito (naive, *w*Mel-infected *n* = 6; DENV-infected, dual-infected *n* = 5). Grey ellipses represent 95% confidence interval.

Interestingly, principal component analyses (PCA) conducted based on these two sets of lipids as variables reveal that, while one infection status separated the samples along one dimension, samples were further segregated along a second dimension corresponding with the other infection status (Fig. 1). In both PCAs, dual-infected samples tended to cluster apart from samples of other infection status. We take this as evidence for interaction between the effects of DENV3 × *Wolbachia*. However, we found no lipids with abundances significantly modulated by the interaction effect of DENV3 × *Wolbachia* after stringent multiple testing criteria (analysis of variance (ANOVA), BH-adjusted *p* < 0.05). Given that we are looking for sets of candidates to be further empirically tested, we removed the multiple testing criteria (ANOVA, unadjusted *p* < 0.05) and found 218 lipids modulated by DENV3 × *Wolbachia* interaction. We proceeded to investigate this latter set of lipids for potential biological significance, with 31 lipids putatively identified at MSI level 2 and 110 lipids at MSI level 3. Of note, no overlap occurred between the sets of lipids significantly enriched by DENV3 and *Wolbachia* mono-infections, which would have indicated shared requirement and thus competition for lipids.

### Lipids altered by DENV3 infection

**Fig. 2:**
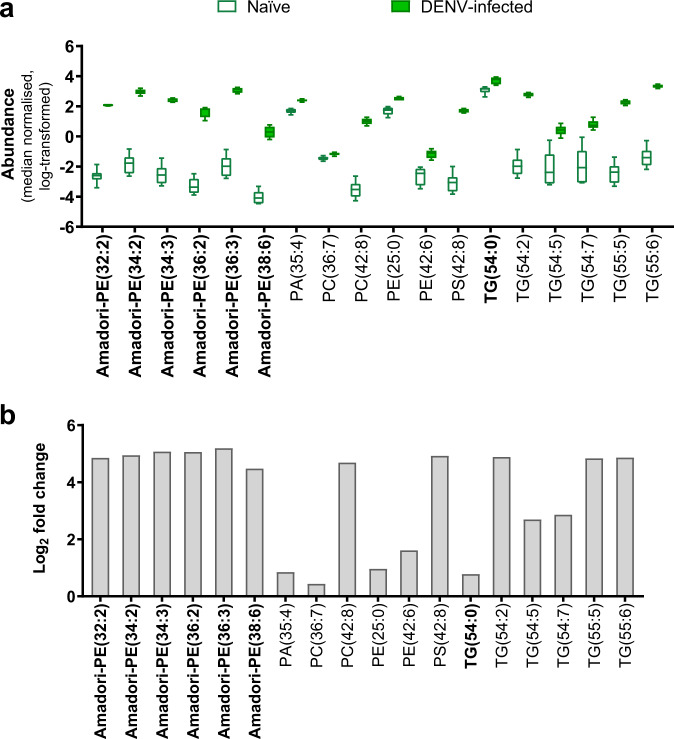
DENV3 mono-infection increased the abundances of lipids. Lipid profiles of naive mosquitoes were compared with DENV3 mono-infected mosquitoes. **a** Boxplots show absolute abundances (median-normalized, natural log-transformed) in which boxes extend from 25th to 75th percentile, middle line denotes median, and whiskers denote minimum and maximum values (naive *n* = 6; DENV3-infected *n* = 5). **b** Bar graphs depict log2 fold changes of representative lipids with others listed in Supplementary Data 1. Species names in bold were identified to MSI level 2 while others were identified to MSI level 3. PE phosphoethanolamine, PA phosphatidic acid, PC phosphocholine, PI phosphoinositol, PS phosphoserine, TG triacylglyceride. The O-prefix denotes an alkyl ether linkage. Numbers in parentheses denote the total number of carbons: double bonds present in the molecule.

To find lipids modulated in response to DENV3 infection alone, we compared lipid abundances between DENV3 mono-infected and naive mosquitoes (Fig. 2). Most of the putatively identified virus-modulated lipids are glycerophospholipids (Supplementary Data 1)^33–35^. Consistent with the study by Perera et al.^29^ of DENV2 infection in mosquito cells, many of the altered glycerophospholipids possessed fatty acids with greater levels unsaturation. PC(42:8) and PS(42:8) subclasses were particularly enriched, showing log_2_ fold changes > 4 (Supplementary Data 1). Unsaturated fatty acids are known to influence the curvature of membranes and are implicated in the formation of the membrane structures induced in cells during infection with DENVs^21,36^.

We observed the enrichment (log_2_ fold changes > 4) of nine glycated phosphoethanolamines (Amadori-PEs) (MSI level 2) in DENV3 mono-infected mosquitoes. Although first discovered in human blood^37^, we conclude that the Amadori-PEs detected here were synthesized in vivo as these mosquitoes were never given a bloodmeal. DENV3 mono-infection also significantly enriched five species of triacylglycerols (TGs) (MSI level 3)—a lipid class from the glycerolipids category—one of which was verified with MS/MS spectra as TG(54:0) (MSI level 2) (Fig. 2) (Supplementary Data 1).

### Lipids modulated by *w*Mel infection

**Fig. 3:**
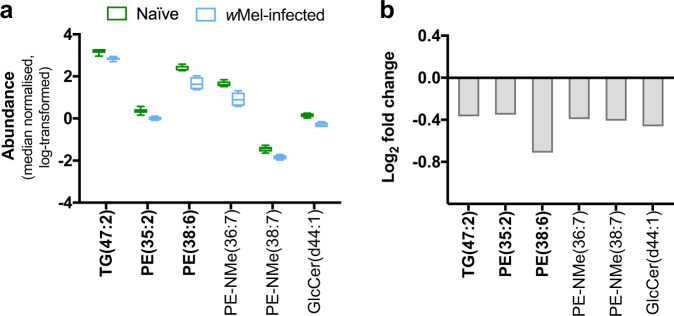
*Wolbachia* mono-infection depleted a small number of lipids. Lipid profiles of naive mosquitoes were compared with *w*Mel mono-infected mosquitoes. **a** Boxplots show absolute abundances (median-normalized, natural log-transformed) in which boxes extend from 25th to 75th percentile, middle line denotes median, and whiskers denote minimum and maximum values (*n* = 6). **b** Bar graphs depict log2 fold changes. Species names in bold were identified to MSI level 2 while others were identified to MSI level 3. TG triacylglyceride, PE phosphoethanolamine, GlcCer glucosylceramide. The -NMe prefix denotes the presence of a methyl group with a nitrogen atom attached. Numbers in parentheses denote the total number of carbons: double bonds present in the molecule.

Lipid abundances in *w*Mel mosquitoes were compared to those of naive mosquitoes in the absence of DENV3 infection. *w*Mel mono-infection perturbed a small number of lipids, causing mild depletions for a TG molecule, four PEs and a glucosylceramide (GlcCer) (Fig. 3 and Supplementary Data 2). Two of the four PE subclasses were methylated (PE-Nme) (MSI level 3), which suggests they are intermediates in the biochemical conversion of PEs to phosphocholines (PCs)^38^. The lipid GlcCer (d44:1) (MSI level 3) is a sphingolipid. This lipid group has roles in regulatory signaling that controls apoptosis, cell cycle, endocytosis, and vesicular trafficking^39,40^.

### Lipids modulated by DENV3 × *Wolbachia* interaction

**Fig. 4:**
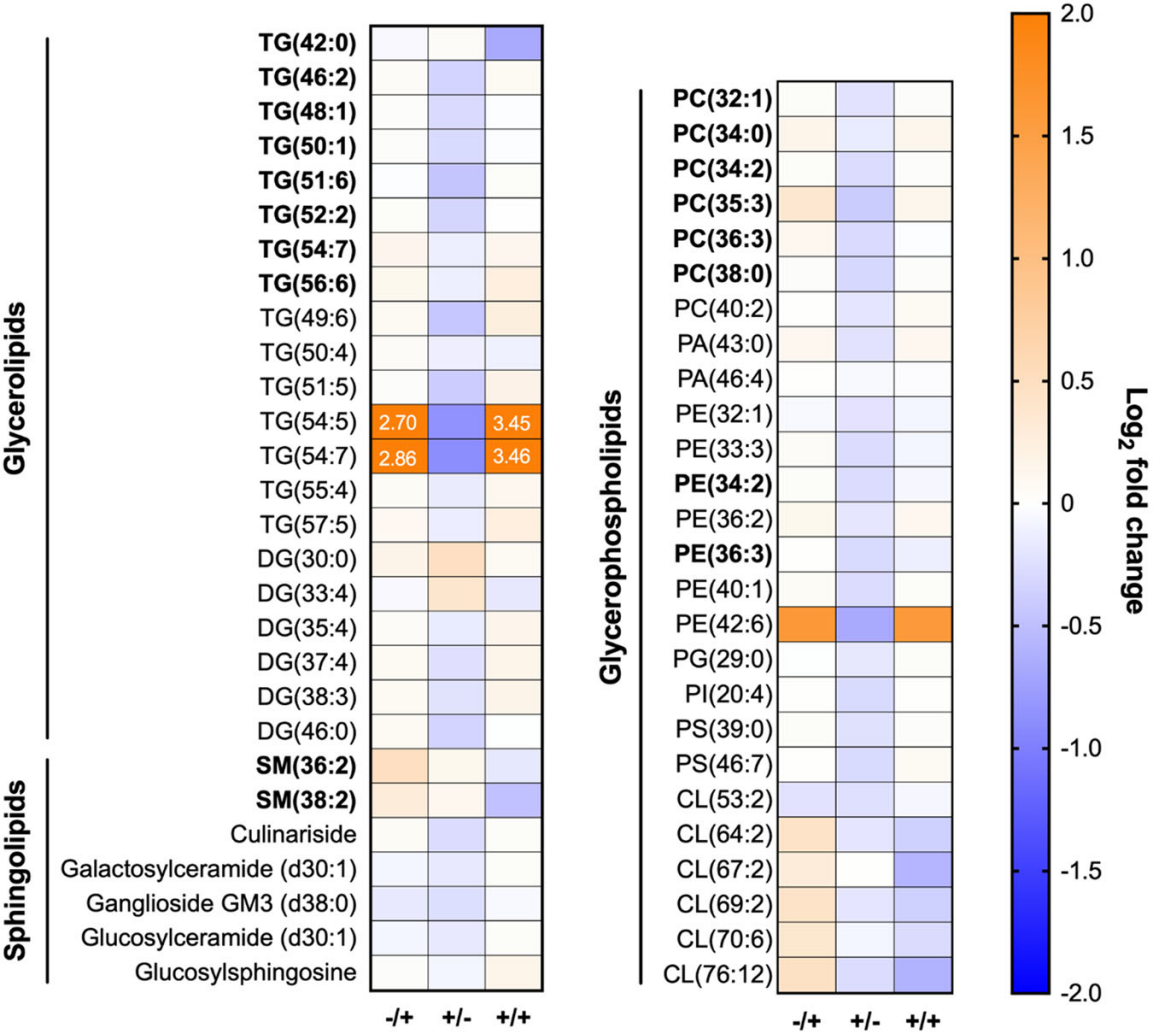
Fold changes of lipids significantly modulated by DENV3 × *Wolbachia* interaction relative to naive mosquitoes. −/+ denotes DENV3 mono-infected mosquitoes. +/− denotes *Wolbachia* mono-infected mosquitoes. +/+ denotes dually-infected mosquitoes. Legend bar shows log2 fold change. Values out of range are indicated in the relevant cells. Species names in bold were identified to MSI level 2 while others were identified to MSI level 3 only. TG triacylglyceride, DG diacylglyceride, SM sphingomyelin, GM3 monosialodihexosylganglioside, CE cholesterol ester, PC phosphocholine, PA phosphatidic acid, PE phosphoethanolamine, PG phosphoglycerol, PI phosphoinositol, PS phosphoserine, CL cardiolipin. Numbers in parentheses denote the total number of carbons: double bonds present in the molecule. Only a subset of representative lipids is depicted in this graph. The complete dataset can be found in Supplementary Data  3.

Since we observed no overlap among the lipids perturbed by DENV3 or *Wolbachia* mono-infection, we hypothesized that *Wolbachia* may be antagonizing DENV3 replication by interfering with virus-induced modulation of the host metabolomic environment. Surprisingly, dual-infected mosquitoes generally resembled DENV mono-infected mosquitoes (Fig. 4 and Supplementary Data 3). Among differentially modulated lipids, DENV3 mono-infection tended to cause mild enrichment of most lipids except for three, TG(54:5), TG(54:7), and PE(42:6), which were enriched strongly (log_2_ fold change > 1). In contrast, *Wolbachia* alone induced mostly mild depletions (log_2_ fold change < 1 or >−1)(Supplementary Data 3). In dual-infected mosquitoes, most lipids tended to exhibit modulations in the same direction as in DENV3 mono-infected mosquitoes. However, we were particularly interested in those modulated differentially by DENV3 depending on *Wolbachia* presence as they were more likely to underpin *Wolbachia*-induced virus blocking. Two lipid classes—sphingomyelins (SM) and CLs—were enriched by DENV3 mono-infection but depleted by dual-infection (Fig. 4). SMs and other classes of sphingolipids are synthesized by reversible enzymatic conversions within the sphingolipid biosynthesis network, which begins with the de novo synthesis of ceramides from serine and palmitoyl-CoA in the ER^41^. As two SMs, SM(36:2) and SM(38:2), showed a complementary direction of perturbation to the other four species of sphingolipids in response to DENV3 mono-infection, this may be indicative of a preference for SMs over other classes of sphingolipids for virus replication. CLs are a member of glycerophospholipids specifically localized in mitochondrial or bacterial membranes. Eleven out of 12 putatively identified CLs were elevated in DENV3 mono-infected mosquitoes but depleted in dual-infected mosquitoes (Supplementary Data 3).

### Selection of candidate genes for dsRNA knockdown

Based on the lipidomic data and known roles in cellular function, we targeted further investigation on the TG, SM, and CL lipid classes. To validate the biological importance of these lipid classes in DENV3 and *Wolbachia* replication, we sought to disrupt their biosynthesis pathways through knockdown of key enzymes using dsRNA. Based on KEGG metabolic pathways, the genes *DGAT1*, *SPT1*, and *CRLS* were selected as ideal candidates for knockdown (Table 1) as the strategic locations of these enzymes within their respective pathways would maximize the effect of knockdown on the targeted lipid classes.

**Table 1:** Candidate genes for dsRNA knockdown and their roles within lipid biosynthesis pathways.

Lipid profile-derived candidate genes			
Gene name	Gene ID	Lipid pathway	Function
Diacylglycerol O-acyltransferase 1 (DGAT1)	AAEL001204	aag00561: glycerolipid metabolism	Converts diacylglycerols (DGs) to triacylglycerols (TGs)
Serine palmitoyltransferase-1 (SPT1)	AAEL010610	aag00600: sphingolipid metabolism	Rate-limiting enzyme of the sphingolipid synthesis pathway
Cardiolipin synthase (CRLS)	AAEL014198	aag00564: glycerophospholipid metabolism	Converts phosphatidylglycerols (PGs) to cardiolipins (CLs)

Literature-derived candidate genes			
Gene name	Gene ID	Reported relevance	Function
Phospholipase D3 (PLD3)	AAEL003651	Knockdown of this gene reduced *Wolbachia* density in *Drosophila* cells^20^	Hydrolyzes PCs and CLs to PAs
Pancreatic lipase-related protein 2 (PNLIP)	AAEL014551	Upregulated in *Ae. fluviatilis* when infected by the *w*Flu *Wolbachia* strain^42^	Hydrolyzes TGs into DGs and free fatty acids
Very low density lipophorin receptor (VLDLR)	AAEL012251	Upregulated in A. aegypti fat body when infected with *Plasmodium* *gallinaceum*^47^	Associates with TGs or DGs for transport through the hemocoel
Cardiolipin synthase (CRLS)	AAEL014198	Downregulated in Aag2 cells challenged with DENV and *Enterobacter cloacae*^43^	Produces CLs from a PG and a DG molecule

**Fig. 5:**
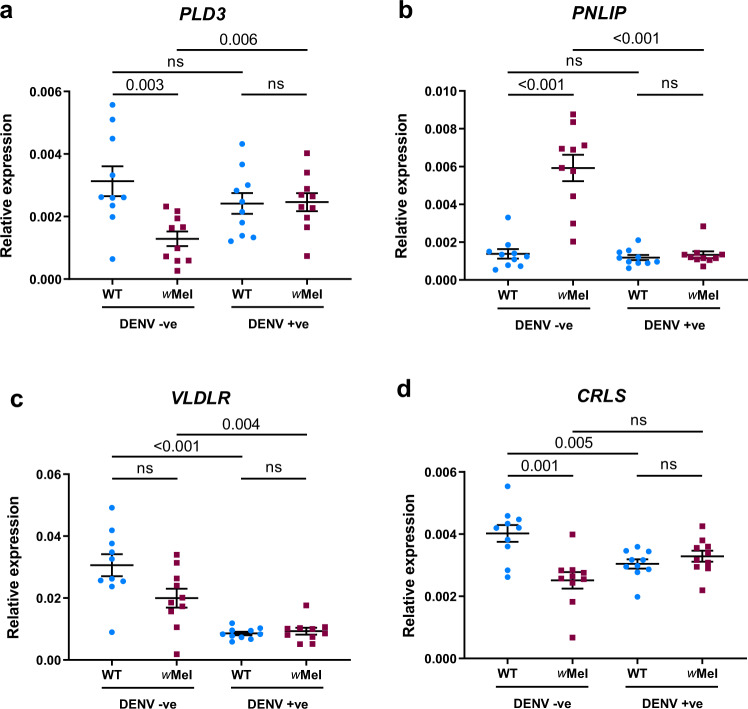
Relative expression levels of literature-derived candidate genes. Gene expression was quantified in WT (blue circles) and *Wolbachia*-infected (maroon squares) mosquitoes by qRT-PCR at 7 days post-injection with sterile media or DENV3. Expression levels of **a** *phospholipase D3*, **b** *pancreatic lipase-related protein 2*, **c** v*ery low density lipophorin receptor*, and **d** *cardiolipin synthase* were normalized to a housekeeping gene, *RpS17*. Mean and standard error are shown in graphs. Each data point represents one mosquito (*n* = 10 per condition). *P* values of post hoc comparisons are shown (Student’s two-sided *t* test with Bonferroni correction).

To complement our lipid profile data, we looked in published transcriptomic and genetic studies for genes related to lipid metabolism affected by infection in *Drosophila* or *Aedes* systems^20,42–48^ We compiled a list of 13 genes with altered expression levels associated with the infection of *Wolbachia* or other pathogens (Supplementary Table 1). To identify those that were relevant to our study, we used quantitative real-time polymerase chain reaction (qRT-PCR) to screen for genes whose expression levels are altered in our mosquito colonies as a result of DENV3 or *Wolbachia* infection. Four genes exhibited differential responses to DENV3 infection depending on *Wolbachia* presence (Table 1): *phospholipase D3* (*PLD3*), *pancreatic lipase-related protein 2* (*PNLIP*), *very low density lipophorin receptor* (*VLDLR*), and *Cardiolipin synthase* (*CRLS*) (ANOVA, DENV3 × *Wolbachia* interaction *p* < 0.05) (Table 2). *PLD3* was downregulated while *PNLIP* was elevated in *Wolbachia* mono-infected mosquitoes relative to other infection status. *VLDLR* was downregulated by DENV infection in WT and *w*Mel mosquitoes. *CRLS* was downregulated by both DENV3 and *Wolbachia* mono-infections (Fig. 5). Notably, the functions of these four genes are associated with TGs and CLs (Table 1).

**Table 2:** Two-way analyses of variance (ANOVAs) on gene expression of literature-derived candidate genes in response to *Wolbachia* or DENV3 infection.

Gene	Factors	*F* value	*p* value
*Phospholipase D3* (*PLD3*)	*Wolbachia*	6.779	0.013
	DENV3	0.443	0.510
	DENV3 × *Wolbachia*	7.408	0.010
*Pancreatic lipase-related protein 2* (*PNLIP*)	*Wolbachia*	37.017	<0.001
	DENV3	38.470	<0.001
	DENV3 × *Wolbachia*	32.450	<0.001
*Very low density lipophorin receptor* (*VLDLR*)	*Wolbachia*	4.192	0.048
	DENV3	45.946	<0.001
	DENV3 × *Wolbachia*	5.572	0.024
*Cardiolipin synthase* (*CRLS*)	*Wolbachia*	8.066	0.007
	DENV3	0.211	0.649
	DENV3 × *Wolbachia*	15.713	<0.001

### CRLS knockdown affects DENV3 and *Wolbachia* loads

To test how reduced expression of our candidate genes would affect the replication of DENV3 and *Wolbachia*, double-stranded RNAs (dsRNAs) targeting these genes were injected intrathoracically into WT and *w*Mel mosquitoes in parallel with sterile medium or DENV3. As a negative control for dsRNA treatment, mosquitoes were injected with dsRNA targeting a partial sequence for green fluorescent protein (GFP). Knockdown was confirmed in naive mosquitoes through comparison of anti-gene of interest (GOI) dsRNA-treated mosquitoes to their control-treated counterparts at 4 days post-injection. dsRNA knockdown successfully reduced gene expression for *PLD3*, *PNLIP*, *VLDLR*, and *CRLS* but not for *DGAT1* or *SPT1* (Supplementary Table 2). Expression levels in dsRNA-treated mosquitoes are shown in Supplementary Fig. 9.

**Fig. 6:**
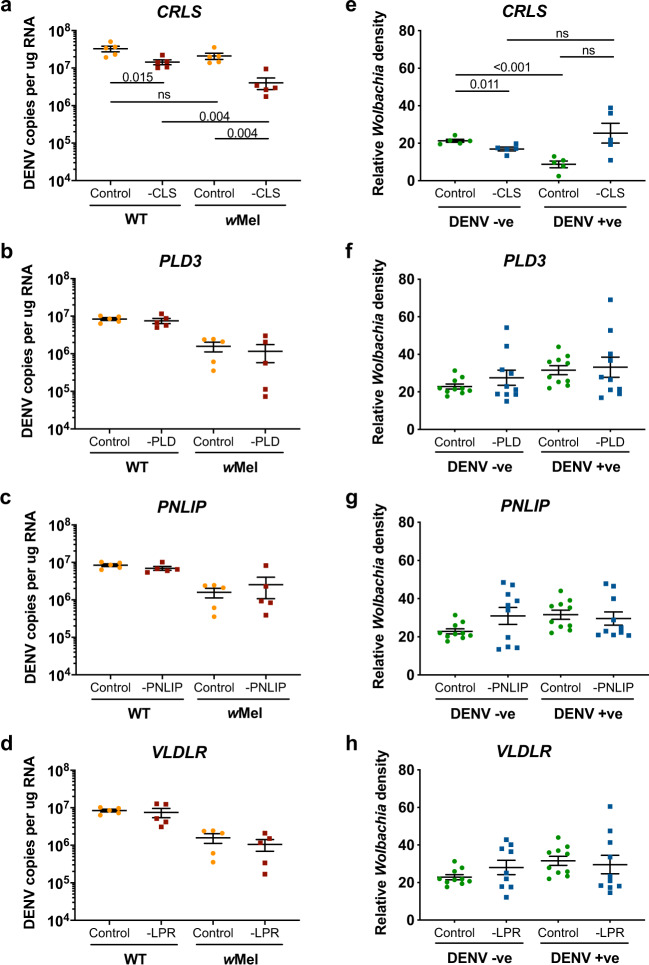
Effect of dsRNA treatment on DENV3 loads and *Wolbachia* density. To validate the functional role of certain lipid classes on DENV3 replication, WT and *w*Mel mosquitoes were injected with DENV3 in parallel with either anti-GOI dsRNA (orange circles) or control dsRNA (maroon squares). **a**–**d** Viral loads were quantified by qRT-PCR as genome copies per μg of extracted RNA at 4 days post-injection (*n* = 5 mosquitoes per condition). To validate the functional role of certain lipid classes on *Wolbachia* replication, *w*Mel mosquitoes were treated with either control dsRNA (green circles) or with anti-GOI dsRNA (blue squares), with and without DENV3. **e**–**h** Relative *Wolbachia* densities were quantified by qRT-PCR as the copy number ratio of the *Wolbachia* gene *TM513* to the mosquito gene *RpS17* in genomic DNA at 4 days post-injection (*CRLS*: *n* = 5 mosquitoes per condition; *PLD3*, *PNLIP*, *VLDLR*: *n* = 10 mosquitoes per condition). Mean and standard error are shown in graphs. Each data point represents one mosquito. *P* values are shown where post hoc comparisons were made (Student’s two-sided *t* test with Bonferroni correction).

At 4 days post-injection, virus genome copies were quantified by qRT-PCR from total RNA and ANOVAs were performed to assess the impact of dsRNA knockdown on virus replication (Table 3). In all cases, there was a negative effect of *Wolbachia* infection on DENV3 loads as expected. There was a significant main effect of dsRNA treatment only with anti-*CRLS* dsRNA, whereby viral copy numbers were reduced (Fig. 6). This suggests the function of *CRLS*, and therefore the ready availability of CLs, is beneficial for virus replication. There were no significant effects of dsRNA × *Wolbachia* interaction on DENV loads.

To assess whether dsRNA treatment impacted *Wolbachia* replication, relative *Wolbachia* densities were quantified from mosquito DNA at 4 days post-injection. Here, once again there was a main effect of dsRNA treatment and an effect of DENV3 × dsRNA interaction only in the case of anti-*CRLS* dsRNA (Table 3). Post hoc comparisons revealed anti-*CRLS* dsRNA treatment reduced *Wolbachia* density in DENV3-free mosquitoes. DENV3 infection significantly reduced *Wolbachia* density in control dsRNA-treated mosquitoes (Fig. 6), which would suggest that DENV3 co-infection hampers *Wolbachia* replication, although this effect was not observed in experiments with other dsRNAs, making it difficult to interpret how the abundance of CLs contribute to the blocking phenotype. In dual-infected mosquitoes, *Wolbachia* density apparently increased when treated with anti-*CRLS* dsRNA, although this was not statistically significant after Bonferroni multiple test correction is applied (α = 0.0125, *p* = 0.017). As this was accompanied by a decrease in DENV3 load (Fig. 6) in the same mosquitoes, it is possible that *CRLS* function is important for both DENV3 and *Wolbachia* replication, but less so for *Wolbachia* in dual-infected mosquitoes.

**Table 3:** Two-way analysis of variance testing the effects of *Wolbachia* and dsRNA treatment on DENV3 loads.

Effects of *Wolbachia* and dsRNA treatment on DENV3 loads			
Gene	Factors	*F* value	*p* value
*Phospholipase D3* (*PLD3*)	*Wolbachia*	70.911	<0.001
	dsRNA	0.671	0.425
	dsRNA × *Wolbachia*	0.081	0.780
*Pancreatic lipase-related protein 2* (*PNLIP*)	*Wolbachia*	35.546	<0.001
	dsRNA	0.078	0.783
	dsRNA × *Wolbachia*	1.666	0.215
*Very low density lipophorin receptor* (*VLDLR*)	*Wolbachia*	33.976	<0.001
	dsRNA	0.398	0.537
	dsRNA × *Wolbachia*	0.029	0.868
*Cardiolipin synthase* (*CRLS*)	*Wolbachia*	9.272	0.008
	dsRNA	23.497	<0.001
	dsRNA × *Wolbachia*	0.037	0.851

Effects of DENV3 and dsRNA treatment on *Wolbachia* density			
*Phospholipase D3* (*PLD3*)	DENV3	3.052	0.089
	dsRNA	2.563	0.118
	dsRNA × DENV3	0.099	0.755
*Pancreatic lipase-related protein 2* (*PNLIP*)	DENV3	0.214	0.647
	dsRNA	1.665	0.205
	dsRNA × DENV3	1.124	0.296
*Very low density lipophorin receptor* (*VLDLR*)	DENV3	1.737	0.196
	dsRNA	1.697	0.201
	dsRNA × DENV3	0.044	0.834
*Cardiolipin synthase* (*CRLS*)	DENV3	0.516	0.483
	dsRNA	4.586	0.048
	dsRNA × DENV3	13.453	0.002

## Discussion

DENVs and *Wolbachia* have reduced genomes and are unable to synthesize the complete suite of lipids they require for replication^49,50^. Thus, infection by either would cause extensive disruptions in host lipid composition and metabolism. Competition for key lipids is popularly proposed as one of the underlying mechanisms of *Wolbachia*-induced virus blocking^11,12,51^. Our study aimed to find metabolomic evidence in support of this. To understand the influence of DENV3 and *Wolbachia* individually on host lipid repertoire, we compared the lipid profiles of mosquitoes bearing DENV3 or *Wolbachia* mono-infection to that of naive mosquitoes. Generally, DENV3 mono-infection greatly increased lipid abundances whereas *Wolbachia* mono-infection caused mild depletions. Although PCAs suggest there is interaction between DENV3 and *Wolbachia* effects, there was no overlap between DENV3- or *Wolbachia*-enriched lipids as would be expected if they were co-opting the same lipids. Thus, our lipid profile data offered little evidence for direct metabolic competition. Several lipids—SMs and CLs, which play roles in signaling pathways and mitochondrial function respectively—exhibited differential DENV3-modulated abundances depending on *Wolbachia* presence. This indicates antagonistic lipid modulation on these lipid classes by the two metabolic parasites, either through suppression of virus-induced modulations or conversion of pro-virus lipids into different pro-*Wolbachia* lipids.

As a result of DENV3 mono-infection, several TG species were strongly elevated. TGs are the main constituents of lipid droplets—fat storage organelles in the cell surrounded by a phospholipid membrane^52^. In *A. aegypti*, TGs are also the form in which lipids are transported throughout the hemolymph in association with lipophorin proteins^53^. This elevation concurs with lipid droplet growth and increase in TG abundance documented in mosquito and mammalian cells infected with DENVs^43,54^. Barletta et al.^43^ posits that TG enrichment supports the increased cellular energetic demands of mounting an immune response against pathogens. However, as TGs are the primary form of lipid storage, this is inconsistent with the notion of increased energy production from lipid stores. TG levels should decrease as a result of increase lipolysis rate to liberate free fatty acids to feed the β-oxidation pathway, which results in ATP generation. Indeed, this occurs in mammalian cells infected with DENVs, where increased rates of β-oxidation is necessary for viral replication^25^. This is accompanied by reduced lipid droplet size indicative of lipid droplet-specific autophagy^25^. Interestingly, lipid droplet biogenesis is also connected to the immune response, as activation of Toll and IMD pathways triggers lipid droplet biogenesis in mosquitoes and mammals^43,55^. These pathways also activate the expression of antimicrobial proteins with antiviral activity^56^. It is possible that lipid droplets serve a bioactive role in mosquito innate immunity, instead of being passive energy reservoirs. On the other hand, as the capsid protein of DENVs associates with lipid droplet membranes in mammalian and mosquito cells, these organelles may aid in virion particle assembly^54^. Given these contradictory reports and hypothesized functions of TG enrichment and lipid droplet growth in response to infection with DENVs, these events possibly constitute a double-edged sword where they are favorable to both virus replication and host antiviral immunity.

Another notable feature of DENV3 mono-infection is the detection of Amadori-PEs. These molecules are the product of a single hexose sugar group conjugated to the amine group of PEs under hyperglycemic conditions^57^. Such conditions are consistent with the increase in cellular glucose uptake for glycolysis observed during infection with DENVs in human cells^58^. Typically reported in diabetic blood plasma and organs, Amadori-PEs are more susceptible to peroxidation and in turn promotes the peroxidation of other lipid classes^59^. In mosquito cells, this could exacerbate the oxidative damage associated with viral infection^60^. Amadori-PEs are formed spontaneously without an enzyme catalyst^61^ and so they are most likely a by-product of infection processes. Their presence in our dataset is a metabolic biosignature that suggests infection-associated intracellular hyperglycemia in mammalian cells also occurs in infected mosquitoes. In macrophages, Amadori-PEs are linked to TG accumulation^62^. A similar association can be seen in our DENV3-modulated dataset, although whether these two phenomena are occurring within the same tissue or cells cannot be determined.

Our DENV3-modulated lipid profiles also recapitulated the findings of a previous lipidomic study by Perera et al.^29^ in which glycerophospholipids high in polyunsaturated fatty acids were enriched as a result of DENV2 infection in mosquito cells. These polyunsaturated fatty acids favor the formation of highly fluid and leaky membranes that are ideal for the assembly and function of viral replication complexes^21,24^.

The effects of *w*Mel modulation on the *A. aegypti* lipidome were marginal in comparison to DENV3 despite the limited capacity of *w*Mel for lipid metabolism^50^ and the reported hijacking of intracellular membranes to form protective vesicular envelopes around the endobacteria^20,63^. The *w*Mel genome suggests they rely on amino acids as an energy source rather than on lipids or carbohydrates. As such, we expected to find modulations mainly in lipid classes associated with structural membranes, namely PCs and PEs. Although there is a paucity of quantitative investigations into the effects of *Wolbachia* on host metabolism, several key studies had shaped our expectations. Molloy et al.^28^ found pronounced perturbations by the strain *w*Mel in *A*. *albopictus* Aa23 cells. PCs and PEs were depleted along with CERs, SMs, and DGs, while PGs and PIs were enriched^28^. Geoghegan et al.^64^ investigated the proteomic changes associated with *w*MelPop infection in *A. aegypti* Aag2 cells and reported an increase in sphingomyelinase abundance—the catalyst enzyme that converts SM into CER^39^—yet SMs were unaltered by *Wolbachia* mono-infection in our study. This may be due to the dilution of any tissue-specific alterations by studying the whole organism metabolome, thus reducing the overall significant changes observed. As metabolic responses to viral infection can differ greatly between cell culture systems and whole organisms^26^, this likely applies to *Wolbachia* infection as well. Being maintained in liquid media, cells in culture might respond more instantaneously to metabolic perturbations.

In dual-infected mosquitoes, glycerolipids (TGs, DGs, and monoacylglycerides) were altered by DENV3 infection differentially, depending on *Wolbachia* presence. This may be indicative of perturbations in cellular energy homeostasis and lipolytic activity^65^. In energy deficient conditions, lipolysis occurs where TGs are mobilized from lipid droplets to produce free fatty acids to feed the β-oxidation pathway in the mitochondria, where breakdown of fatty acids lead to ATP production^66,67^. This process would deplete TGs (triple fatty acid chains) and increase the presence of DGs (double fatty acid chains) and monoacylglycerides (single fatty acid chain). In our study, several TG species were depleted by *Wolbachia* mono-infection, accompanied by elevation in some DG species (Supplementary Data 3). This would be consistent with increased conversion of lipid stores into energy. These alterations did not appear in dual-infected mosquitoes. Instead, lipolysis levels in them were comparable to DENV mono-infected mosquitoes, suggesting that modulations favorable for virus replication prevail in these mosquitoes.

Cholesterol has an important role in DENV3 replication as evidenced by reduced virus replication, assembly, and virus-induced mortality in mice treated with Lovastatin, a cholesterol-lowering drug^68–70^. However, excess cholesterol is detrimental to virus infection^71^. Geoghegan et al.^64^ found that the *Wolbachia* strain *w*MelPop strongly inhibited DENV replication in *A. aegypti* cells and higher levels of esterified cholesterol was reported in the system. The authors suggest that intracellular cholesterol trafficking and homeostasis is disrupted in *Wolbachia*-infected cells. We did not observe any cholesterols among the lipids differentially affected by the DENV3 × *Wolbachia* interaction to support the hypothesis that *Wolbachia* is unfavorably modulating cholesterol availability for DENV3 replication under our experimental conditions and methods.

DENV3 × *Wolbachia* interaction has a notable effect on the abundance of CLs. Most were elevated in DENV3 mono-infected mosquitoes yet depleted in *Wolbachia* mono-infected or dual-infected mosquitoes. This is particularly interesting as CLs are a class of glycerophospholipids uniquely found in bacterial membranes or in the matrix side of mitochondrial membranes of eukaryotic cells. In the mitochondria, CLs bind and stabilize components of the electron transport chain complexes, maintaining mitochondrial function^72^ and inhibiting cytochrome c-induced apoptosis^73^. Studies have shown how infection by DENVs causes alterations in mitochondrial morphology, which are favourable for virus replication^31,32^. To our knowledge, the is the first empirical link between CLs and DENV or *Wolbachia* proliferation. The differential perturbation of CLs and its biological role within the mitochondria provides a basis for further investigation. Here, we validated the role of CL using dsRNA-mediated knockdown of its synthase enzyme, *CRLS*. This resulted in decreased DENV3 loads regardless of *Wolbachia* presence, suggesting that DENV3 relies on an optimum availability of CLs for efficient replication. *Wolbachia* replication also suffered from *CRLS* knockdown in the absence of DENV3 infection.

CL depletion may impact DENV3 replication in two non-mutually exclusive ways: apoptosis regulation and energy metabolism. Under normal cellular conditions, CLs support ATP-generating oxidative phosphorylation in the mitochondria by anchoring components of the electron transport chain complexes in the inner mitochondrial membrane^73^. The interaction between cytochrome c, a key component of the electron transport chain, with CLs plays a particularly important role in mitochondria-induced apoptosis regulation^73^. Under conditions of high oxidative stress, peroxidation of CLs cause the dissociation and release of cytochrome c into the cytoplasm. Cytoplasmic cytochrome c triggers a caspase-dependent signaling pathway leading to apoptosis^73–75^. Apoptosis is detrimental for virus replication, which benefits from prolonged cell survival during early infection stages. There is some evidence that DENVs and other flaviviruses stave off apoptosis through interactions with cytochrome c-independent signaling pathways^76–78^. Thus, a high abundance of CLs would benefit virus proliferation by suppressing the initiation of an apoptotic cascade via cytochrome c. In the context of energy metabolism, lack of CLs to stabilize components of the oxidative phosphorylation machinery can result in decreased ATP generation necessary for viral amplification, assembly, and egress^72,79,80^. DENV infection causes increase in glycolysis^58^, lipolysis^81^, β-oxidation^25^ and accumulation of its substrate, acyl-carnitines^26^. All these processes facilitate the mitochondrial production of ATP, suggesting energy production in the host is a crucial factor of virus replication. In our experiment, *CRLS* knockdown might be compromising virus accumulation by increasing the rate of apoptosis of infected cells or slowing down virus replication through ATP shortage. The importance of the mitochondria for DENV replication is further demonstrated by reports that virus-induced mitochondrial elongation in mammalian cells facilitates DENV infection, either by suppressing activation of interferon responses^31^ or increasing energy production^32^.

*CRLS* knockdown-induced decrease in cellular ATP generation is also likely to impact *Wolbachia* replication. Although the *w*Mel genome contains the necessary pathways to obtain energy from amino acids^50^, the lack of genes encoding components of the oxidative phosphorylation process suggests a reliance on host mitochondria to carry out this final step of ATP generation. In addition, as CLs are also a constituent of bacterial membranes, *CRLS* knockdown may have led to reduced membrane synthesis for *Wolbachia*. Indeed, *CRLS* knockdown reduced *Wolbachia* density in mono-infected mosquitoes.

The effect of *CRLS* knockdown under a dual-infected state was less clear. It is interesting that *CRLS* knockdown led to a greater decrease in DENV3 load in the presence of *Wolbachia*, though this may be partly explained by a slight rise in *Wolbachia* density within the same mosquitoes, as there is some evidence for a positive correlation between *Wolbachia* density and blocking strength^82–84^. For *Wolbachia*, reduced DENV3 replication as a result of *CRLS* knockdown may free up host resources, thus conferring *Wolbachia* an advantage to offset the detrimental effects of CL depletion on its own replication efficiency.

The results of our study should be considered with several caveats. Using whole mosquitoes, we can only report on global body-wide trends of lipid modulation. Given the variation in *Wolbachia* density across mosquito tissues^85^ and the temporal dynamics of viral tissue tropism beginning from the midgut infection^86^, we can expect to find tissue-specific perturbations of the host lipid repertoire. Further research may wish to explore whether some tissues are more malleable to modulation by DENVs than others or if *Wolbachia*-favoring modulations correlate with local bacterium densities. Having key functions in immune response and energy mobilization, the fat body is of immediate interest in this regard. It should also be considered whether DENV3-induced lipid modulation following oral infection vary temporally. Being infected via intrathoracic injection to ensure delivery of a standardized amount of virus across individual mosquitoes and treatments, the modulations reported in this study may reflect only mid- or late-stage infections. Here, the need for consistency outweighed a focus on natural feeding. As the effects of *Wolbachia*-induced blocking extend beyond the midgut infection stage^7^, trends in lipid modulation underpinning blocking would still be captured in our study. In addition, it is hard to determine whether these lipidomic modulations are a result of active modulation by *Wolbachia* or DENV3 as opposed to a passive consequence of mono- or dual-infection. For example, DENV3 infection is associated with both lipid droplet growth^43^ and induction of ER stress^87^. However, ER stress itself can trigger lipid droplet formation^88^. Care must be taken when concluding that DENV3 drives growth in lipid droplets as some lipid modulation may also be mosquito-driven in response to viral infection. Another caveat is the lack of lipidomic validation of the effects of dsRNA knockdown. Our results suggested that high expression of *CRLS* is beneficial for DENV3 or *Wolbachia* replication. Whether knockdown of *CRLS* results in depletion of CL abundance and, in turn, reduced virus and bacterium replication, remains to be demonstrated. To further test our proposed model, pharmacological manipulation of CL abundance are needed to verify its effects on virus and symbiont replication rates and the amount of oxidative damage generated under such conditions. That said, there is often a disconnect between transcriptomics, proteomics, and metabolomics studies investigating the same biological phenomenon. Lastly, although we found that knockdown of *PLD3*, *PNLIP,* and *VLDLR* did not affect DENV3 loads or *Wolbachia* density, the associated lipid classes may still be of importance. There may be redundancies in the biosynthetic pathways such that the functions of these targeted enzymes can also be performed by other enzymes. Alternatively, the strength of knockdown may be insufficient to produce a notable metabolic effect. In general, more functional studies are needed to elucidate *Wolbachia* lipid requirements in the *A. aegypti* host. Although there have been many studies on the effects of lipid manipulation on DENV replication, similar studies for *Wolbachia* are sparse. Our work and that of Molloy et al.^28^ has demonstrated that *Wolbachia*-induced modulation is mild relative to DENVs. As it stands, it appears DENV3 remodels the host lipid repertoire more aggressively than *Wolbachia* does, suggesting a greater reliance on host lipids for survival.

To conclude, this study in *A. aegypti* mosquitoes reveals DENV3 mono-infection caused remarkable increases in abundance for a diverse range of lipids, while *Wolbachia w*Mel infection caused only mild perturbations in a few. We present metabolic evidence of intracellular events associated with DENV3 infection—lipid droplet growth and hyperglycemia. We found limited evidence for competition between DENV3 and *Wolbachia* for the same host lipids. This points to indirect lipid antagonism by *Wolbachia* against DENVs through suppression of pro-viral lipid modulations or active conversion of pro-virus lipids into different pro-*Wolbachia* lipids. DENV3-induced modulations appeared to dominate in dual-infected mosquitoes except for a small number of SM and CL lipids. Lastly, we highlight CLs as potential DENV3 host factors and validated their importance for efficient DENV3 replication. Our work uncovers a part of the still growing picture of metabolic remodeling induced by DENVs and *Wolbachia* infections and offers new research directions to pursue.

## Methods

### Mosquito rearing

We used an *A. aegypti* mosquito colony infected with the *Wolbachia* strain *w*Mel and a naturally *Wolbachia*-free colony, designated *w*Mel and wild type (WT), respectively. As a control for *Wolbachia* infection, natural WT mosquitoes were used over antibiotic-cured *w*Mel mosquitoes to avoid confounding our results with potential cross-generational side effects of antibiotic treatment or persistent *Wolbachia*-induced lipid modulations. Both colonies were established from eggs collected from Cairns (Australia) from within or outside of *Wolbachia* release zones. The *Wolbachia* infection status of both lines were confirmed as per methods described in Yeap et al.^89^. All mosquitoes were reared under standard insectary conditions of 25 °C, 65% relative humidity, and on a 12:12 h light:dark cycle. Larvae were raised on fish food pellets (Tetramin Tropical Tablets, Melle, Germany) while adults were provided with 10% sucrose solution to feed ad libitum. All experiments were conducted using 6–8-day-old mosquitoes.

### Virus culture and titration with plaque assay

We utilized a low passage number DENV3, isolated from a patient during an outbreak in Cairns in 2008/2009^90^, the same region where the mosquito colonies for this study were obtained. Virus was propagated in *A. albopictus* C6/36 cells maintained on RPMI 1640 medium (Life Technologies, Carlsbad, CA) supplemented with 2% heat-inactivated fetal bovine serum (FBS) (Life Technologies), 1% GlutaMAX (Life Technologies), and 25 mM HEPES buffer (Sigma-Aldrich, St. Louis, MO) at 26 °C. At 7 days post-infection, virus was harvested by centrifugation of the cell culture medium at 3200 × *g* at 4 °C. The viremic supernatant was stored at −80 °C until use for intrathoracic microinjections or titration with plaque assay. For plaque assay, six tenfold dilutions of virus stock were prepared and inoculated onto confluent BHK cells in 24-well plates, maintained in DMEM medium supplemented with 10% FBS (Life Technologies) and 1% GlutaMAX (Life Technologies). Following inoculation, virus was allowed to incubate in a 1:1 mixture of 1% carboxymethylcellulose:DMEM medium supplemented with 4% FBS (Life Technologies) and 2% GlutaMAX (Life Technologies) for 5 days at 37 °C with 5% CO_2_. Cells were then fixed with 3.7% paraformaldehyde in PBS, washed and stained with crystal violet to visualize plaques. Virus stock was determined to be at a concentration of 6 × 10^6^ plaque forming units/mL.

### Virus infection and lipid profiling sample collection

WT and *w*Mel mosquitoes were injected intrathoracically using the Nanoject II microinjector (Drummond Scientific, Broomall, PA) with 69 nL of DENV3 virus (a total of 400 plaque forming units delivered per mosquito) or sterile non-supplemented RPMI medium as a negative control. Intrathoracic injection was our preferred method of infection as it allows delivery of a controlled and consistent amount of virus into each mosquito. While more similar to natural events, oral infection via bloodmeal introduces unwanted variability in virus input and dietary lipid intake as the amount of imbibed viremic blood can differ greatly among mosquitoes. Post-injection, mosquitoes were returned to standard rearing conditions. To confirm DENV3 infection, a single leg from each virus-injected mosquito was collected at 7 days post-injection and assayed for the presence of virus, which indicated a disseminated infection^91^. Only mosquitoes with disseminated infections were collected for lipid extraction to ensure uniformity of DENV3 infection stage.

Viral loads in samples were assayed through qRT-PCR. To prepare samples for qRT-PCR, each mosquito leg was homogenized in 50 μL of extraction buffer (10 mM Tris pH 8.2, 1 mM EDTA, 50 mM NaCl, supplemented with 1.25% (v/v) proteinase K (Bioline, Memphis, TN)). This mixture was then incubated for 5 min at 56 °C, followed by 5 min at 95 °C^89^.

For each infection status (Supplementary Fig. 1), six individual mosquitoes were collected for lipid profiling. Total lipids were extracted from whole mosquitoes using a modified protocol based on Folch’s method^92^. Briefly, each mosquito was homogenized using a TissueLyser II (Qiagen, Hilden, Germany) in 400 μL of chilled 100% methanol mixed with 800 μL of chilled 100% chloroform. At this step, two internal standards, D31-16:0-18:1 PE and 19:0-19:0 PC were added to homogenized samples. After sonication for 30 min, the mixture was centrifuged to separate supernatant from debris. The supernatant was then evaporated under vacuum using a SpeedVac concentrator (Thermo Fisher Scientific, Waltham, MA) to obtain dry lipid pellets for subsequent analysis.

### Liquid chromatography–mass spectrometry (LC–MS)

Lipid samples were analyzed by injecting 2 μL of extracted sample reconstituted in 100 μL methanol:butanol (1:9, v/v) into an Agilent 1200 series LC system in-line with an Agilent 6550 quadrupole time-of-flight mass spectrometer (Agilent Technologies, Santa Clara, CA) operated in positive ionization mode. Two reference masses (*m/z* 121.050873 and 922.009798) were used to ensure mass accuracy. LC was performed using a Kinetex Phenomenex EVO C18 column (100 mm × 2.1 mm × 1.7 μm) according to analytical methods adapted from Castro-Perez et al.^93^. Briefly, Solvent A consisted of H_2_O:acetonitrile (4:6, v/v) with 10 mM ammonium formate and solvent B consisted of acetonitrile:isopropanol (1:9, v/v) with 10 mM ammonium formate. Lipids were eluted over a 25-min gradient of solvents A:B (68:32, v/v) to solvents A:B (3:97, v/v) at a flow rate of 0.26 mL/min. Column oven temperature was set to 45 °C throughout analysis time. LC–MS data files were converted to mzXML format using ProteoWizard software^94^. Feature detection, peak picking, peak area measurement, and retention time alignment were performed with XCMS in R according to Smith et al.^95^. Peak annotation was conducted using CAMERA^96^. Parameters for LC–MS and MS/MS data acquisition, data processing and peak annotation are specified in Supplementary Table 3. From the LC–MS dataset, one sample from each of *Wolbachia* mono-infected group and dual-infected group were excluded from the LC–MS dataset because a PCA (not shown) of all lipid features revealed these two samples to be outliers relative to their respective treatment groups.

Abundance data based on area-under-curve were median-normalized as described in Wang et al.^97^ and natural log-transformed to account for heteroscedasticity. Calculations of mean, standard error, fold changes, and statistical analyses were conducted on Stata (version 15). Significantly altered lipids as a result of DENV3 or *Wolbachia* mono-infection were found by comparing mono-infected mosquitoes to naive WT mosquitoes using Student’s two-sided *t* tests. *P* values were adjusted for false discovery using the BH method^98^. To confirm *t*-test results and to visualize effects of DENV3 or *Wolbachia* mono-infection, PCAs were conducted based on sets of statistically significant lipids using ggplot2 package (version 3.3.0) on R (version 4.0.0). Lipids affected by the interaction of DENV3 and *Wolbachia* infections were found by conducting two-way ANOVAs.

Differentially modulated features (*p* < 0.05) were putatively identified by searching mass-to-charge ratio (*m/z*) against the HMDB^99–102^ and LIPID MAPS^103^. [M + H]+, [M + Na]+, and [M + NH4]+ adducts were accounted for in the neutral mass calculation in the positive ionization mode. Features with MS/MS spectra were further interpreted manually and compared with published spectra data^104–107^ (Supplementary Figs. 2–7). In this study, we report metabolite identification levels according to the MSI outlined by Sumner et al.^108^. The MSI level for each differentially modulated feature is specified in the Supplementary Data 1–3.

### Relative quantification of candidate genes expression

We measured gene expression levels to validate candidate genes found from literature search (Supplementary Table 1) and to verify the efficiency of dsRNA knockdowns. Total RNA was isolated from ten mosquitoes of each infection status using TRIzol reagent as per manufacturer’s protocol (Life Technologies). RNA pellets were reconstituted in RNase-free water and treated with DNase I recombinant enzyme (Roche, Basel, Switzerland) according to manufacturer’s protocol. cDNAs were synthesized with SuperScript III Reverse Transcriptase (Invitrogen, Carlsbad, CA) with random primers (Thermo Fisher Scientific) following manufacturer’s protocol. Gene expression was quantified by qRT-PCR with SYBR Green I (Roche, Basel, Switzerland) with the default thermal cycling conditions of the LightCycler 480 instrument II (Roche). Each reaction was set up per manufacturer’s protocol, containing 2 μL of cDNA sample in a total volume of 10 μL. qRT-PCR primers of each candidate genes were designed using Primer-BLAST^109^. Primer sequences are reported in Supplementary Table 1. All target genes were normalized to the housekeeping *A. aegypti* gene *RpS17* (F: 5′-TCCGTGGTATCTCCATCAAGCT-3′, R: 5′-CACTTCCGGCACGTAGTTGTC-3′)^110^. ANOVAs were conducted and post hoc comparisons with Bonferroni multiple test correction were made whenever effect of interaction was statistically significant on IBM SPSS Statistics (version 24).

### Gene knockdown with RNA interference

To create dsRNAs for RNA interference, DNA templates 300–600 bp in length were synthesized using Q5 High-Fidelity DNA Polymerase (New England BioLabs, Ipswich, MA) from *A. aegypti* cDNA using primer pairs designed to target the coding sequences of each candidate gene^111^. Primers were designed using the Primer-BLAST online tool^109^ and were linked with a T7 promoter sequence at the 5′ end (TAATACGACTCACTATAGGGAGACCAC)^112^. PCR product size and purity were verified through gel electrophoresis and isolated with MinElute PCR Purification Kit (Qiagen). Templates were then used to produce dsRNA using the MEGAscript T7 Transcription Kit (Ambion, Foster City, CA) and precipitated with lithium chloride as per manufacturer’s instructions. Purified dsRNA products were diluted to a concentration of 4.35 ng/nL and prepared for microinjection together with either DENV3 virus or sterile medium by mixing in a 1:1 (dsRNA:media) ratio. In total, 138 nL of this mixture was injected into WT and *w*Mel mosquitoes with the Nanoject II microinjector (Drummond Scientific). As a control for dsRNA knockdown, mosquitoes were also injected with dsRNA targeting GFP. Knockdown efficiency was measured using five mosquitoes per condition.

### Virus quantification with qRT-PCR

DENV3 was quantified via qRT-PCR using TaqMan Fast Virus 1-Step Master mix (Applied Biosystems, Foster City, CA) from leg samples homogenized in extraction buffer or total RNA extracted with TRIzol (Life Technologies) from whole mosquito samples. Absolute quantification (number of genome copies) was determined using a standard curve of 10^8^–10^1^ copies of DENV3′ UTR with tenfold dilutions^113^ and standardized by amount of RNA input. Reactions were set up as per the manufacturer’s instructions to contain 2 μL of RNA sample in a total volume of 10 μL, using DENV-specific primers (F: 5′-AAGGACTAGAGGTTAGAGGAGACCC-3′, R: 5′-CGTTCTGTGCCTGGAATGATG-3′, Probe: 5′-FAM-AACAGCATATTGACGCTGGGAGAGACCAGA-BHQ1-3′)^114^. In dsRNA knockdown experiments, DENV3 loads were quantified from five mosquitoes per condition.

### *Wolbachia* density quantification with qRT-PCR

DNA was extracted from mosquitoes in dsRNA experiments using TRIzol (Life Technologies) as per manufacturer’s protocol. Both RNA and DNA were extracted from DENV3-injected *w*Mel mosquitoes. From DNA samples, *Wolbachia* density was measured as the number of the single copy *WD0513* (F: 5′-CAAATTGCTCTTGTCCTGTGG-3′, R: 5′-GGGTGTTAAGCAGAGTTACGG-3′, Probe: 5′-Lc640-TGAAATGGAAAAATTGGCGAGGTGTAGG-Iowablack-3′) gene relative to the housekeeping gene *RpS17* (F: 5′-TCCGTGGTATCTCCATCAAGCT-3′, R: 5′-CACTTCCGGCACGTAGTTGTC-3′, Probe: 5′-FAM-CAGGAGGAGGAACGTGAGCGCAG-BHQ1-3′) in a duplex qRT-PCR using LightCycler 480 Probes master mix (Roche)^89^. In dsRNA knockdown experiments, *Wolbachia* densities were quantified from five mosquitoes for *CRLS* and ten mosquitoes for all other genes.

### Statistics and reproducibility

Statistical analyses were conducted on IBM SPSS Statistics (version 24), Stata (version 15), or R (version 4.0.0) as indicated above. The sample sizes for LC–MS and gene expression studies were informed by pilot experiments to verify the amount of variation to be expected between individual mosquitoes. Methodology and biological materials are disclosed as much as possible but if required, further information can be obtained by contacting from the corresponding author. All mosquitoes used in a given experiment were hatched at the same time and raised under the same controlled conditions.

### Reporting summary

Further information on research design is available in the Nature Research Reporting Summary linked to this article.

## Supplementary information

Supplementary Information

Description of Additional Supplementary Files

Supplementary Data 1

Supplementary Data 2

Supplementary Data 3

Supplementary Data 4

Supplementary Data 5

Supplementary Data 6

Supplementary Data 7

Supplementary Data 8

Reporting Summary

Peer Review File

Editorial Policy Checklist

## Data Availability

Data supporting the findings of this study are available within the accompanying Supplementary files. Source data underlying plots shown in figures are provided in Supplementary Data 4–8. All other data, if any, are available upon reasonable request.
