# Supplementary material for: Dengue virus dominates lipid metabolism modulations in Wolbachia-coinfected Aedes aegypti
Source: Commun Biol. 2020 Sep 18;3:518. doi: 10.1038/s42003-020-01254-z (PMC7501868; doi:10.1038/s42003-020-01254-z)
Supplement: Supplementary file 1 — Supplementary Information [file 42003_2020_1254_MOESM1_ESM.pdf]

SUPPLEMENTARY FIGURES

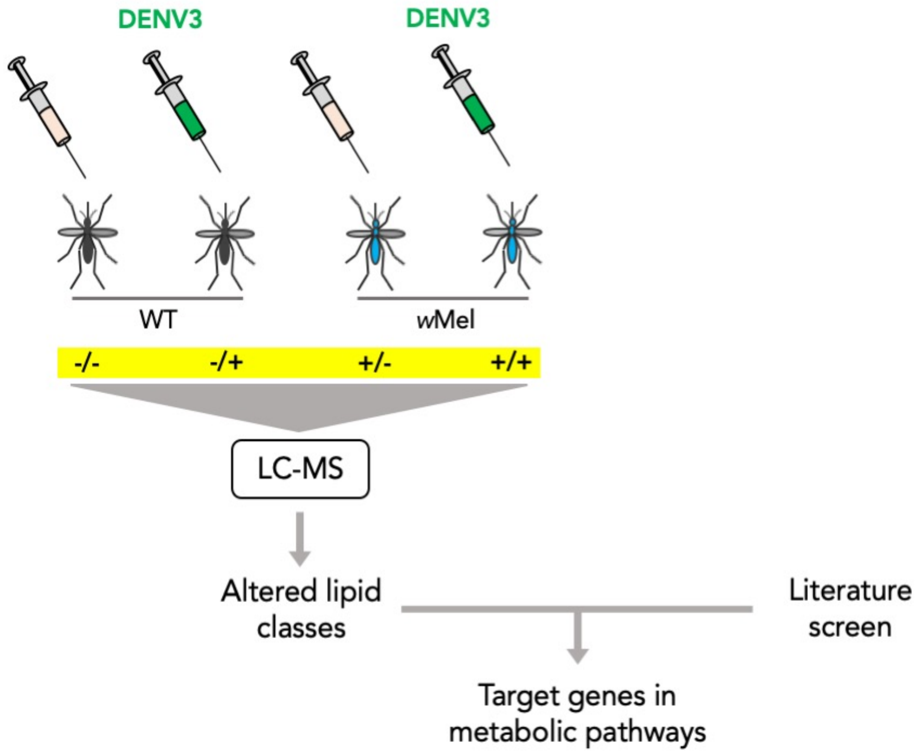

Supplementary Figure 1. Overview of experimental design. Wild-type (WT) and *Wolbachia*-infected (*w*Mel) *A. aegypti* mosquitoes were injected with sterile medium or with DENV3 to create four distinct infection status. Mosquitoes were designated -/-, -/+, +/-, or +/+ (yellow bar) to denote their status of *Wolbachia* or DENV3 infection as follows: -/-, naïve WT mosquito; -/+, DENV3-infected WT mosquito; +/-, uninfected *w*Mel mosquito; +/+, DENV3-infected *w*Mel mosquito. Lipid profiles of these mosquitoes were obtained through LC-MS. Based on analyses of altered lipid classes observed in our dataset and a literature screen of existing studies, we selected lipid metabolism genes to target with dsRNA-mediated knockdown to validate the importance of certain lipid classes in *Wolbachia* and DENV3 infections.

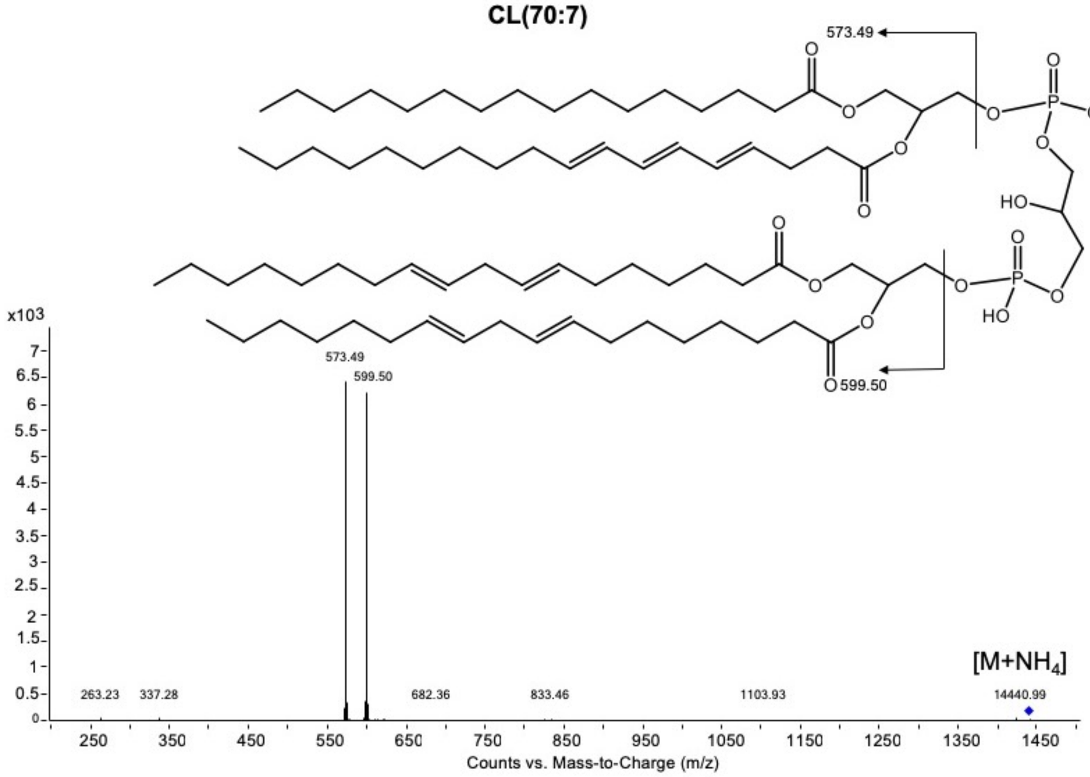

Supplementary Figure 2. MS/MS spectra of Cardiolipin CL(70:7) acquired in positive mode at collision energy 30V. Fatty acid chain and double bond in the structures are arbitrary.

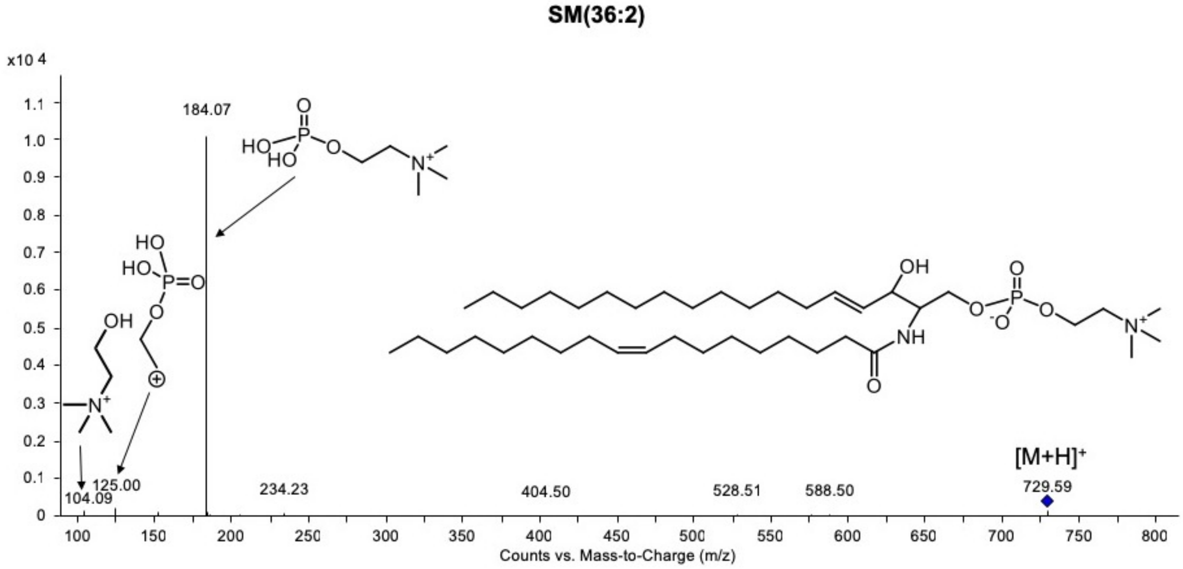

Supplementary Figure 3. MS/MS spectra of Sphingomyelin SM(36:2) acquired in positive mode at collision energy 30V. Fatty acid chain and double bond in the structures are arbitrary.

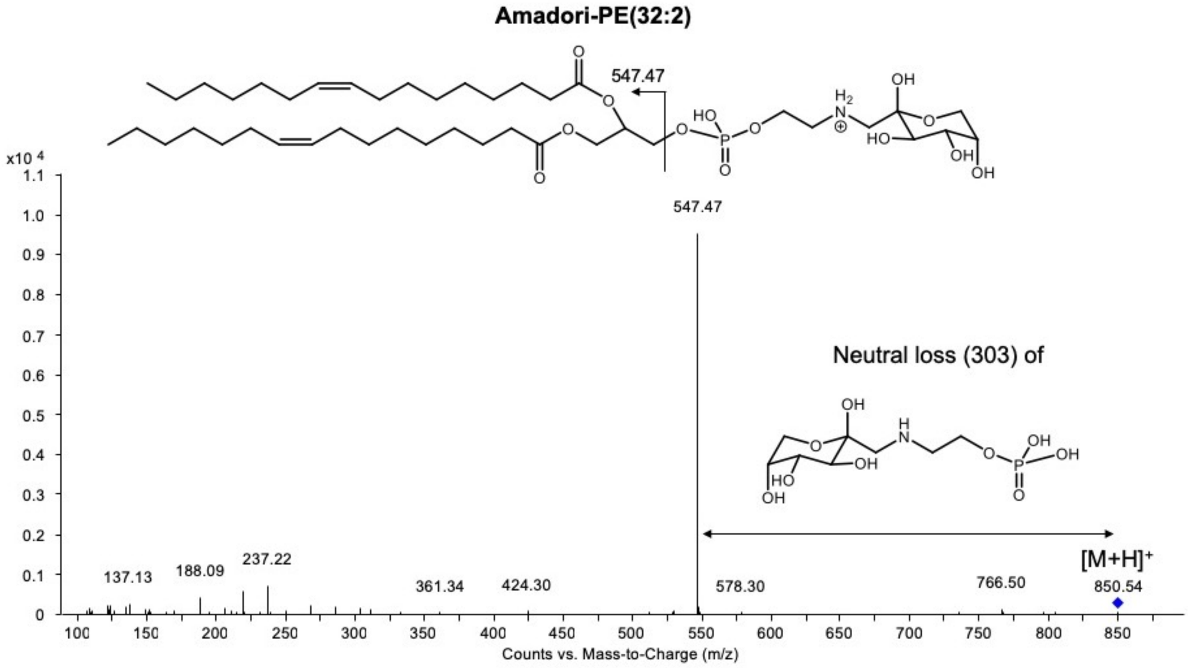

Supplementary Figure 4. MS/MS spectra of Amadori-PE(32:2) acquired in positive mode at collision energy 30V. Fatty acid chain and double bond in the structures are arbitrary.

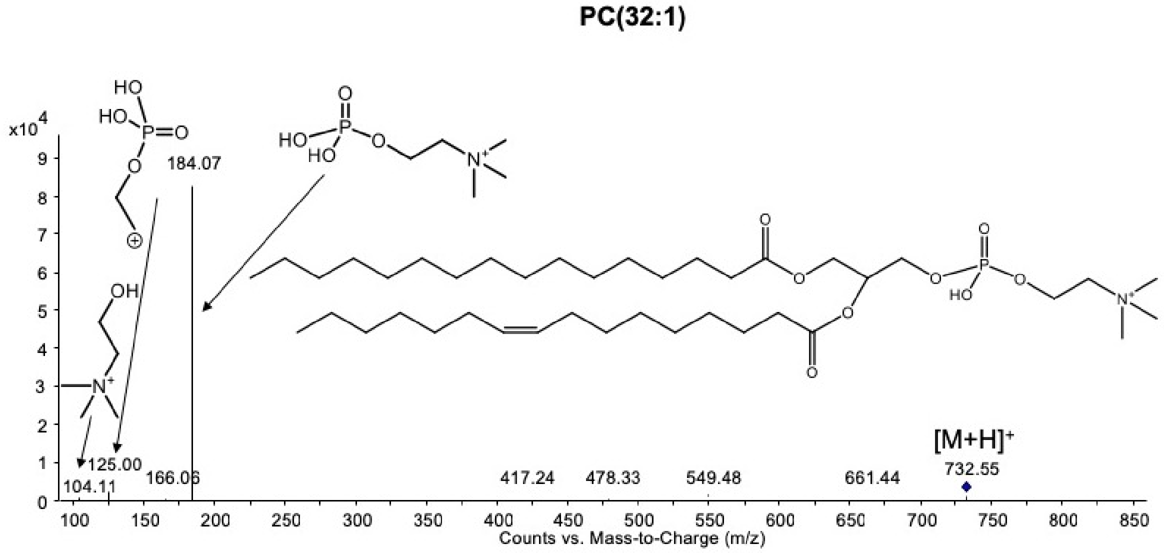

Supplementary Figure 5. MS/MS spectra of Phosphatidylcholine PC(32:1) acquired in positive mode at collision energy 30V. Fatty acid chain and double bond in the structures are arbitrary.

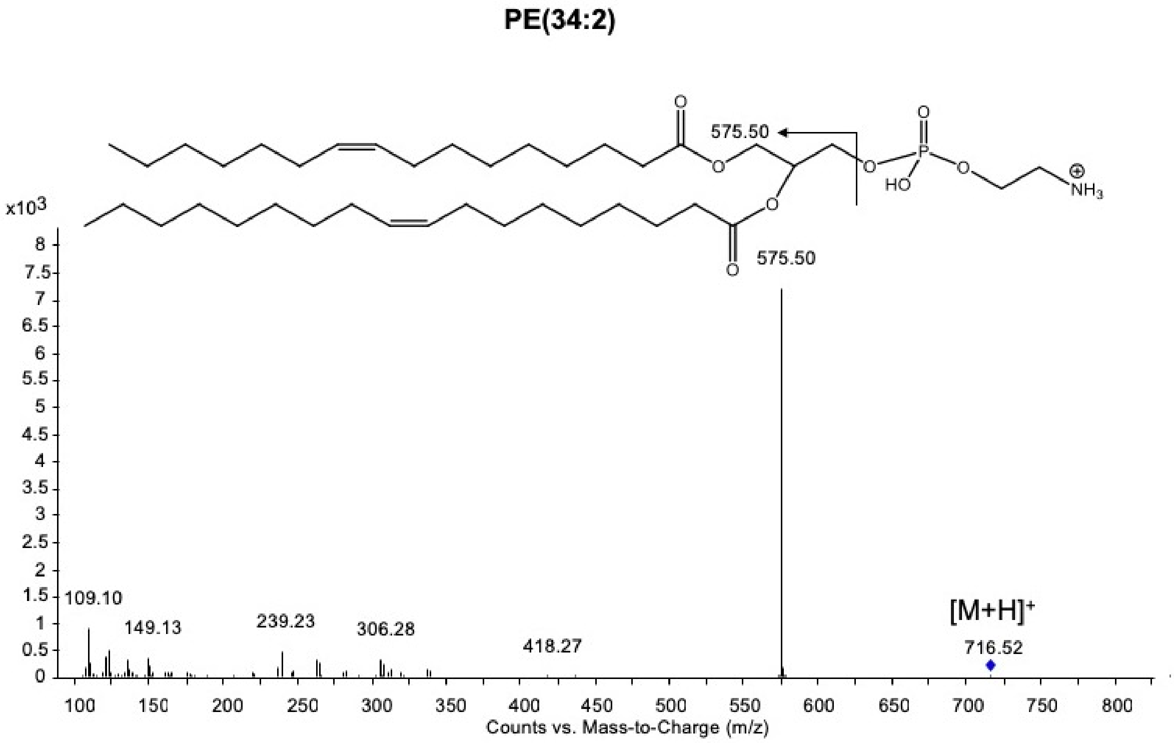

Supplementary Figure 6. MS/MS spectra of Phosphatidylethanolamine PE(34:2) acquired in positive mode at collision energy 30V. Fatty acid chain and double bond in the structures are arbitrary.

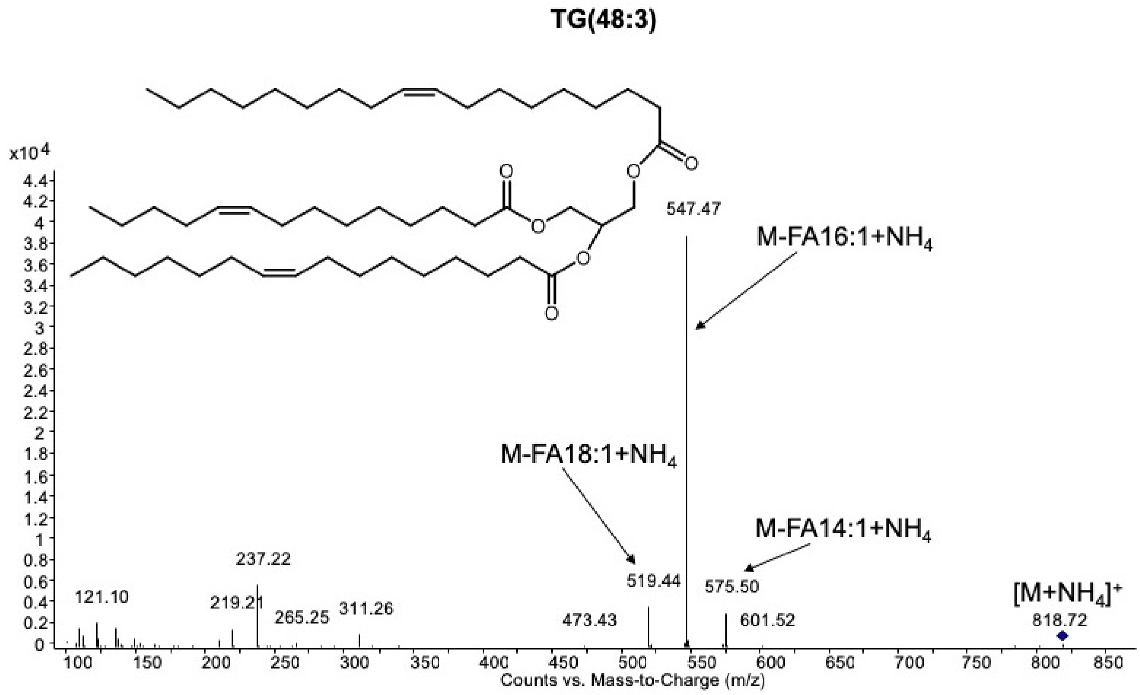

Supplementary Figure 7. MS/MS spectra of Triacylglycerol TG(48:3) acquired in positive mode at collision energy 30V. Fatty acid chain and double bond in the structures are arbitrary.

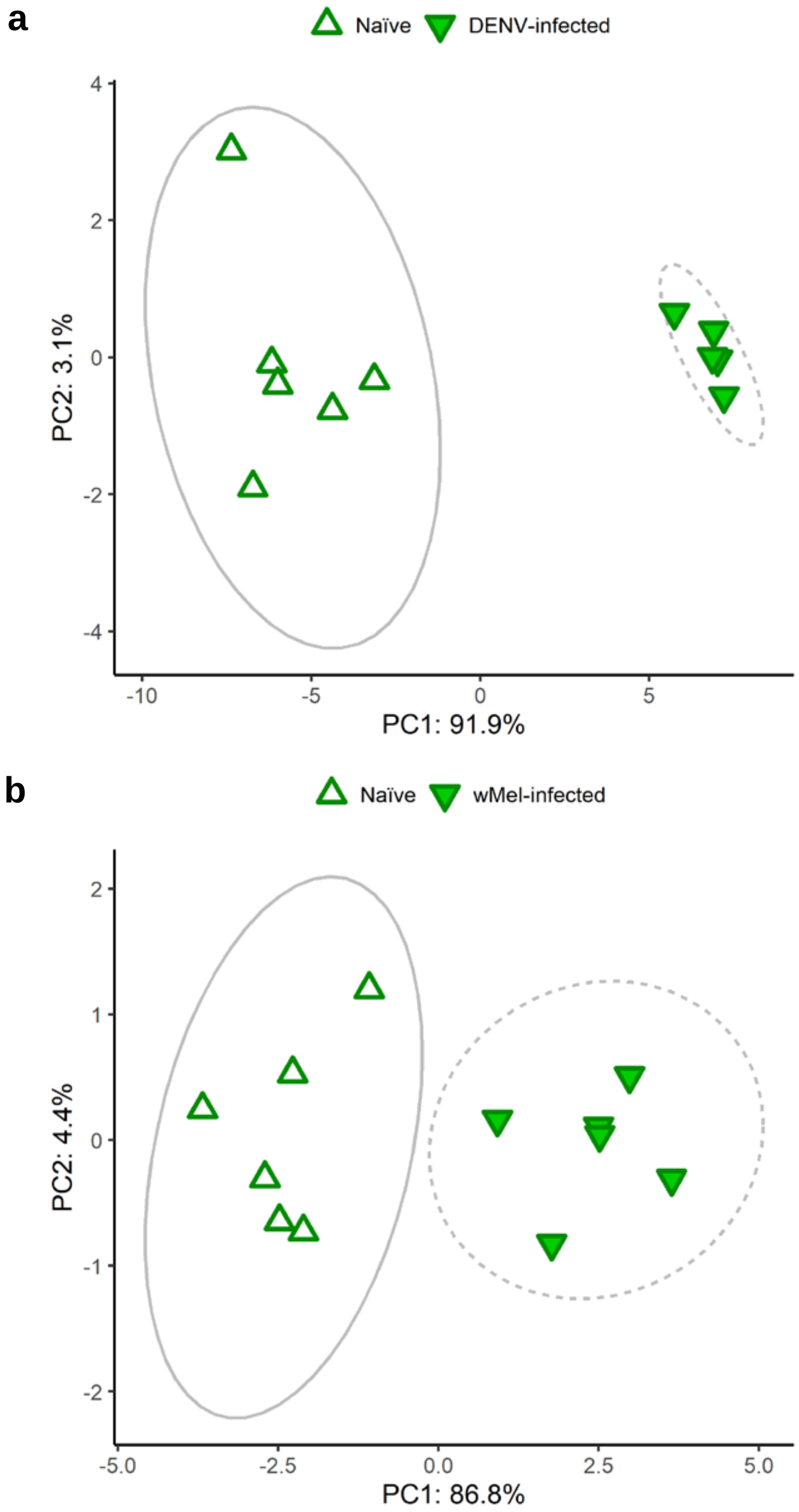

Supplementary Figure 8. Principal components analyses of naïve and DENV3 mono infected mosquitoes (a) and of naïve and *Wolbachia* mono-infected mosquitoes (b). Lipids significantly altered by mono-infection of DENV3 or Wolbachia defines the separation of naïve mosquitoes from their respective infected counterparts. Grey ellipses show boundaries of 95% confidence intervals.

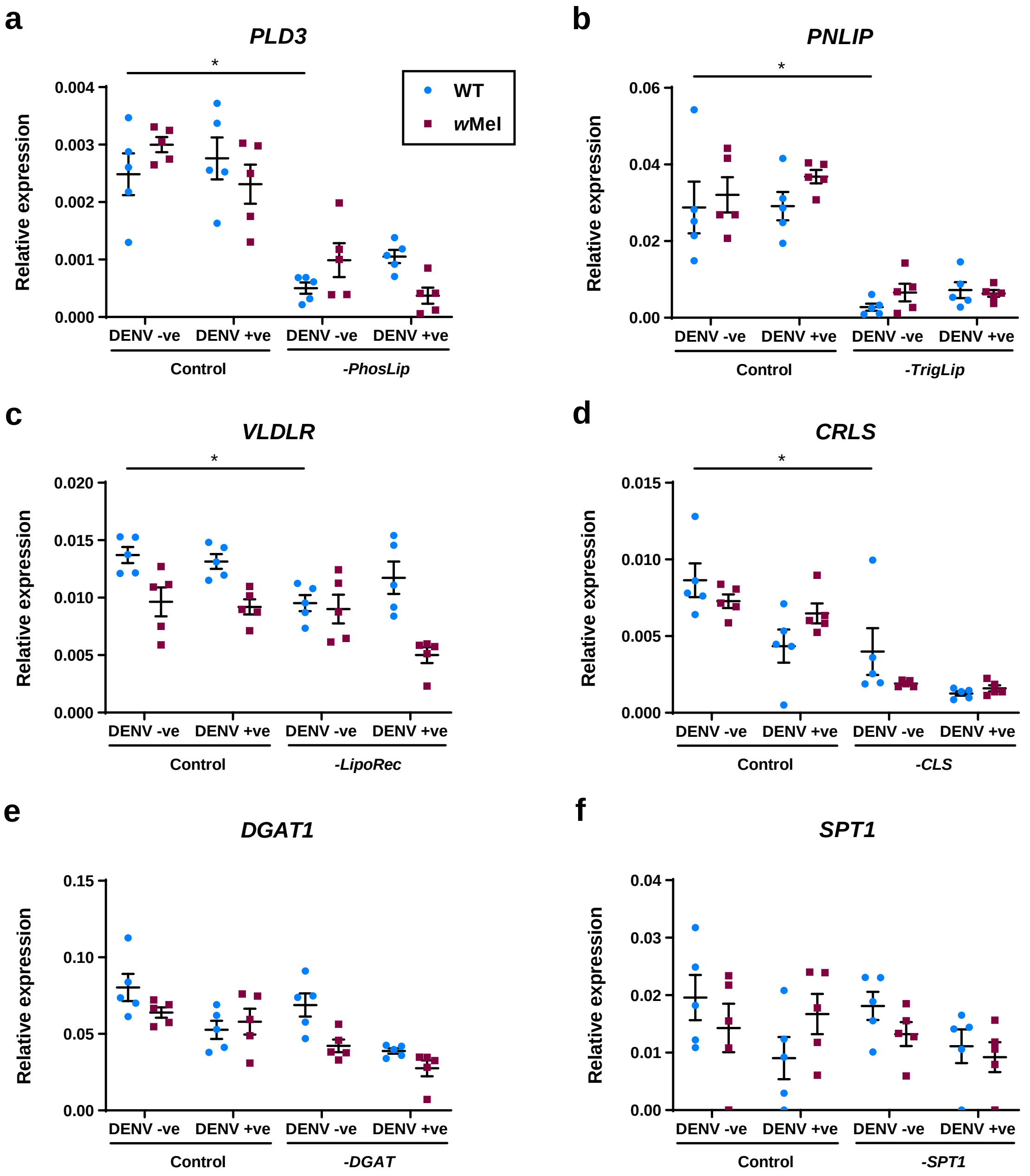

Supplementary Figure 9. dsRNA knockdown effectiveness on expression of candidate gene. Expression levels of six candidate genes were relatively quantified by qRT-PCR and normalized to a housekeeping gene, *RpS17,* in naïve mosquitoes at four days-post-infection with control or target dsRNA. Mean and standard are shown in graphs. Each data point represents one mosquito, n=5 mosquitoes per condition. P-values for statistically significant comparisons are shown (Student's two-sided t-test).

SUPPLEMENTARY TABLES

Supplementary Table 1. Literature-derived candidate genes for dsRNA knockdown.

Gene ID	Gene description	Biological relevance	Reference	Fwd primer (5'-3')
AAEL003651	Phospholipase D, CG43345 orthologue	RNAi screen showed that knockdowns of these genes resulted in decreased *Wolbachia* titre.	White, P. M. *et al.* Reliance of *Wolbachia* on high rates of host proteolysis revealed by a genome-wide RNAi screen of *Drosophila* cells. *Genetics* **205**, 1473-1488 (2017).	Fwd–CGGGAATCCGAGAACCACTT<br>Rev–AGGCACCACGAATCGCTTTA
AAEL004414	Phospholipase D, CG43346 orthologue			Fwd–TACCGAAGAACCCCACTGGA<br>Rev–CTTCTTCCCGGGCATAATTTCAT
AAEL008386	Putative ATP-binding cassette transporter subfamily A, CG1718 orthologue			Fwd–GACCGCTCCTGAGCCAAATA<br>Rev–CACCCAACTTCGCTTTGTGG
AAEL013170	phosphatidylcholine-sterol acyltransferase	Upregulated in *Aedes fluviatilis* infected with the *w*Flu strain of *Wolbachia*, potential host factors.	Caragata, E. P. *et al.* The transcriptome of the mosquito *Aedes fluviatilis* (Diptera: *Culicidae*), and transcriptional changes associated with its native *Wolbachia* infection. *BMC Genomics* **18**, 6 (2017).	Fwd–CGCATTGGTGCAGAATGGTT<br>Rev–GGGGTGTCATCGTTCAAGGT
AAEL014551	pancreatic triglycerol lipase			Fwd–GCGAAATCGGTAAGATGGCG<br>Rev–TAGAGCGGTTCCTGCGAAAG
AAEL012654	pancreatic triglycerol lipase paralogue			Fwd–TGCCTCTCTGGTAGACGTGA<br>Rev–CACAACCCGGCTGTTCTTTG
AAEL000757	anterior fat body protein			Fwd–TTCCCGTGAAAGGAAGGAGC<br>Rev–GTCAGAGGTTCCGTTCCAGG
AAEL014198	Cardiolipin synthase, CG4774 orthologue	Downregulated in Aag2 cells challenged with DENV and *Enterobacter cloacae.*	Barletta, A. B. *et al.* Emerging role of lipid droplets in *Aedes aegypti* immune response against bacteria and dengue virus. *Sci Rep* **6**, 19928 (2016).	Fwd–ACAAGGTGAAGGAACGGGTC<br>Rev–CACAATCCTACCGACGCACA
AAEL008789	Putative apolipophorin-III	Gene with putative role in lipid metabolism during diapause in *Aedes albopictus*.	Reynolds, J. A., Poelchau, M. F., Rahman, Z., Armbruster, P. A. & Denlinger, D. L. Transcript profiling reveals mechanisms for lipid conservation during diapause in the mosquito, Aedes albopictus. *J Insect Physiol* **58**, 966-973 (2012).	Fwd–CGAGGTGCTACCGAACAAGT<br>Rev–TTATGGCTGGTCCTGCTTGG
AAEL012704	Sterol carrier protein-2 like 3 (SCP-2L3)	Knockdown of this gene reduced DENV replication in mosquito Aag2 cells.	Dyer, D. H., Vyazunova, I., Lorch, J. M., Forest, K. T. & Lan, Q. Characterization of the yellow fever mosquito sterol carrier protein-2 like 3 gene and ligand-bound protein structure. *Mol Cell Biochem* **326**, 67-77 (2009).<br>Fu, Q., Inankur, B., Yin, J., Striker, R. & Lan, Q. Sterol Carrier Protein 2, a Critical Host Factor for Dengue Virus Infection, Alters the Cholesterol	Fwd–GAACTAGCAGTCACCAGCAC<br>Rev–GTTTCCTTCGTTGCCACCAG

			Distribution in Mosquito Aag2 Cells. *J Med Entomol* **52**, 1124-1134 (2015)	
AAEL009955	Lipophorin carrier protein orthologue	Upregulated in *Aedes aegypti* during infection by Gram (+) bacteria, fungi and *Plasmodium gallinaceum.*	Cheon, H. M., Shin, S. W., Bian, G., Park, J. H. & Raikhel, A. S. Regulation of lipid metabolism genes, lipid carrier protein lipophorin, and its receptor during immune challenge in the mosquito *Aedes aegypti*. *J Biol Chem* **281**, 8426-8435 (2006).	Fwd–TTCGGCGATTTCAACCTGGA<br>Rev–ACCCTTACCGCTGTTTCCTG
AAEL012251	Lipophorin receptor ovary variant (AF355595)			Fwd–GCACGAATCTAACCCGCATC<br>Rev–CGACGCCGATTGAAGTACGA
AAEL001194	Fatty Acid Synthase 1	Fatty acid synthase protein is redistributed to viral replication sites.	Heaton, N. S. *et al.* Dengue virus nonstructural protein 3 redistributes fatty acid synthase to sites of viral replication and increases cellular fatty acid synthesis. *Proc Natl Acad Sci U S A* **107**, 17345-17350 (2010).	Fwd–TCTGGCTGCCTACTGGAGAG<br>Rev–AGACCCACCGCTGCCATAAG

Supplementary Table 2. dsRNA knockdown successfully reduces expression levels for four gene candidates.

**Gene**	**t-statistic**	**p-value**	**% expression relative to control**
Phospholipase D3 (*PLD3*)	5.268	0.001	20.2
Pancreatic lipase-related protein 2 (*PNLIP*)	3.819	0.005	9.6
Very low density lipophorin receptor (*VLDLR*)	4.203	0.003	69.5
Cardiolipin synthase (*CRLS*)	2.480	0.038	46.1
Diacylglycerol O-acyltransferase 1 (*DGAT1*)	0.978	0.357	85.8
Serine palmitoyltransferase-1 (*SPT1*)	0.313	0.762	92.6

Supplementary Table 3. Parameters for data acquisition, data processing and peak annotation during lipid profiling.

LC-MS data acquisition parameters	
Ionization mode	Positive electrospray
Gas temperature	225°C
Drying gas flow	13 L/min
Nebulizer pressure	20 psig
Capillary voltage	4000 V
Sheath gas flow	12 L/min
Sheath gas temperature	275°C
Fragmentor voltage	360 V
Acquisition rate	2 spectra/sec
Time	500 msec/spectrum (2951 transients/spectrum)
MS/MS data acquisition parameters	
Mode	positive auto Q-TOF MS
Mass range	100–2700 *m/z*
Collision energy	30 eV
Acquisition rate	4 spectra/sec
Time	250 msec/spectrum (1359 transients/spectrum)
Isolation width.	Narrow (~1.3 *m/z*)
XCMS data processing parameters	
Feature detection algorithm	centWave
Ppm error	20 ppm
Maximum peak width	25 seconds
Signal-to-noise threshold	5
Polarity	Positive
RT start	30 seconds
RT end	1440 seconds
CAMERA annotation of isotopes, adducts and group related peaks	
Ppm error	20 ppm
Polarity	Positive
