# Supplementary material for: Dengue virus dominates lipid metabolism modulations in Wolbachia-coinfected Aedes aegypti
Source: Commun Biol. 2020 Sep 18;3:518. doi: 10.1038/s42003-020-01254-z (PMC7501868; doi:10.1038/s42003-020-01254-z)
Supplement: Supplementary file 2 — Description of Additional Supplementary Files [file 42003_2020_1254_MOESM2_ESM.docx]

**Description of Additional Supplementary Files**

Supplementary Data 1. Lipids significantly modulated by DENV3 infection.

Supplementary Data 2. Lipids significantly modulated by *Wolbachia* infection.

Supplementary Data 3. Lipids significantly modulated by DENV3 × *Wolbachia* infections.

Supplementary Data 4. Raw LC-MS abundance data.

Supplementary Data 5. qRT-PCR source data for candidate gene expression.

Supplementary Data 6. qRT-PCR source data for knockdown efficiency.

Supplementary Data 7. qRT-PCR source data for DENV3 titre in dsRNA experiment.

Supplementary Data 8. qRT-PCR source data for *Wolbachia* density in dsRNA experiment.
