# Supplementary material for: Dengue virus dominates lipid metabolism modulations in Wolbachia-coinfected Aedes aegypti
Source: Commun Biol. 2020 Sep 18;3:518. doi: 10.1038/s42003-020-01254-z (PMC7501868; doi:10.1038/s42003-020-01254-z)
Supplement: Supplementary file 12 — Peer Review File [file 42003_2020_1254_MOESM12_ESM.pdf]

This manuscript has been previously reviewed at another Nature Research journal. This document only contains reviewer comments and rebuttal letters for versions considered at Communications Biology.

REVIEWERS' COMMENTS:

Reviewer #1 (Remarks to the Author):

This is very nice work that can further the understanding of role of lipid metabolism in Wolbachia-mediated viral inhibition in mosquito. It can significantly improve the resolution of the current view on how lipid involves in Wolbachia-dengue interactions and has provided convinced and comprehensive evidence against a previous simple hypothesis of direct competition between virus and Wolbachia for lipid to induce viral blocking, but opens a new avenue to study the interaction between Wolbachia-lipid-dengue. The revised version has been improved as suggested and also well addressed my previous concerns on limitation of the dsRNA and intrathoracical injection. I agree that these approaches can be good starting assays with less variation introduced (e.g., lipid in blood can introduces additional variation), providing a base for further studies in a complicated real infection system.

I think that the reviewer 1's comments and suggestions have also been well addressed in this revised version. The amount and quality of the work reported in this manuscript have met the level to be published by Communications Biology.
